# DPP-4 inhibitor linagliptin ameliorates cardiovascular injury in salt-sensitive hypertensive rats independently of blood glucose and blood pressure

**DOI:** 10.1186/s12933-014-0157-0

**Published:** 2014-11-29

**Authors:** Nobutaka Koibuchi, Yu Hasegawa, Tetsuji Katayama, Kensuke Toyama, Ken Uekawa, Daisuke Sueta, Hiroaki Kusaka, MingJie Ma, Takashi Nakagawa, Bowen Lin, Shokei Kim-Mitsuyama

**Affiliations:** Department of Pharmacology and Molecular Therapeutics, Kumamoto University Graduate School of Medical Sciences, 1-1-1 Honjo, Kumamoto, 860-8556 Japan

**Keywords:** Salt-sensitive hypertension, Cardiovascular injury, Oxidative stress, Inflammation, ACE, Pleiotrophic effects

## Abstract

**Background:**

It remains to be elucidated whether dipeptidylpeptidase-4 (DPP-4) inhibitor can ameliorate cardiovascular injury in salt-sensitive hypertension. The present study was undertaken to test our hypothesis that linagliptin, a DPP-4 inhibitor, administration initiated after onset of hypertension and cardiac hypertrophy can ameliorate cardiovascular injury in Dahl salt-sensitive hypertensive rats (DS rats).

**Methods:**

High-salt loaded DS rats with established hypertension and cardiac hypertrophy were divided into two groups, and were orally given (1) vehicle or (2) linagliptin (3 mg/kg/day) once a day for 4 weeks, and cardiovascular protective effects of linagliptin in DS rats were evaluated.

**Results:**

Linagliptin did not significantly affect blood pressure and blood glucose levels in DS rats. Linagliptin significantly lessened cardiac hypertrophy in DS rats, as estimated by cardiac weight and echocardiographic parameters. Linagliptin significantly ameliorated cardiac fibrosis, cardiac macrophage infiltration, and coronary arterial remodeling in DS rats. Furthermore, linagliptin significantly mitigated the impairment of vascular function in DS rats, as shown by the improvement of acetylcholine-induced or sodium nitroprusside-induced vascular relaxation by linagliptin. These cardiovascular protective effects of linagliptin were associated with the attenuation of oxidative stress, NADPH oxidase subunits, p67^phox^ and p22 ^phox^, and angiotensin-converting enzyme (ACE).

**Conclusions:**

Our results provided the experimental evidence that linagliptin treatment initiated after the appearance of hypertension and cardiac hypertrophy protected against cardiovascular injury induced by salt-sensitive hypertension, independently of blood pressure and blood glucose. These beneficial effects of linagliptin seem to be attributed to the reduction of oxidative stress and ACE.

## Introduction

The development of cardiovascular disease begins with risk factors such as diabetes, hypertension, and dyslipidemia, etc. [1]. Strict blood pressure control and lipid control are well known to definitely reduce cardiovascular events in high-risk patients. On the other hand, there has been controversy regarding whether strict glycemic control can reduce cardiovascular disease (macrovascular disease) in type 2 diabetic patients [2-5], although diabetic microvascular complication such as nephropathy, retinopathy, or neuropathy is demonstrated to be reduced by strict glycemic control. This uncertainty about the efficacy of the conventional glucose-lowering therapies on cardiovascular outcome emphasizes the need for novel antidiabetic agents with the benefits in prevention of diabetic macrovascular complication.

Dipeptidylpeptidase 4 (DPP-4) inhibitors are a new class of blood glucose-lowering drug, have been approved for treatment of type 2 diabetes, and take the advantage of having low risk of hypoglycemia and neutral effect on body weight [6-9]. DPP-4 inhibitors inhibit the degradation of incretin hormone, glucagon-like peptide-1 (GLP-1), and consequently prolong the physiologic effect of GLP-1, thereby exerting blood glucose-lowering effect through enhanced physiologically regulated insulin secretion. Interestingly, DPP-4 inhibitors are proposed to likely affect other peptides than GLP-1, since DPP-4 is a multifunctional enzyme and cleaves a number of other substrates than GLP-1, such as neuropeptide, cytokines, and chemokines [6-9]. Previous preclinical studies show that DPP4 inhibitors prevent cardiac diastolic dysfunction [10] and ameliorate glomerulopathy [11] in insulin-resistant Zucker obese rats, ameliorate vascular dysfunction in experimental sepsis [12], reduce myocardial infarct size in cardiac ischemia-reperfusion model [13], or reduce vascular endothelial oxidative stress [14]. However, it remains to be defined whether DPP-4 inhibitor can ameliorate cardiovascular injury beyond blood glucose control. Recent large clinical trials show that alogliptin [15] and saxagliptin [16] failed to reduce cardiovascular events in patients with type 2 diabetes, although the meta-analysis provides evidence that compared to other glucose-lowering therapies, DPP-4 inhibitors may possibly decrease cardiovascular events in patients with type 2 diabetes [7]. There is the possibility that the cardiovascular effects may differ among DPP-4 inhibitors.

Patients with type 2 diabetes often suffer from hypertension, particularly salt-sensitive hypertension. Importantly, salt-sensitive hypertension is associated with greater risk of cardiovascular morbidity and mortality than salt-resistant hypertension [17-21]. Therefore, it is of particular interest to examine whether DPP-4 inhibitor can mitigate cardiovascular injury in salt-sensitive hypertension. To test our hypothesis that DPP-4 inhibition can suppress cardiovascular injury induced by salt-sensitive hypertension, independently of glycemic control and blood pressure control, we examined the effect of linagliptin [22,23], a DPP-4 inhibitor with unique xanthine-based structure, on cardiovascular injury in Dahl salt-sensitive hypertensive rats, a useful model of salt-sensitive hypertension. We obtained the evidence that initiation of linagliptin administration after onset of hypertension and cardiac hypertrophy limited cardiac hypertrophy, fibrosis, inflammation, and vascular dysfunction in salt-induced hypertension independently of blood glucose or blood pressure.

## Methods

### Animals

All procedures were performed in accordance with institutional guidelines for the Care and Use of Laboratory Animals approved by Kumamoto University Graduate School of Medical Sciences. Male Dahl salt-sensitive hypertensive (DS) rats were obtained from Japan SLC Inc (Shizuoka, Japan).

### Experimental protocol

Eight % NaCl diet is known to cause severe hypertension and cardiovascular injury in DS rats, while less than 8% NaCl diet causes mild hypertension and mild cardiovascular injury in DS rats [24,25]. Therefore, in the present study, 8% NaCl diet was used to make a model of salt-sensitive hypertension. DS rats started to be fed an 8% NaCl diet (high-salt diet) from 7 weeks of age, and oral administration of linagliptin to DS rats was initiated from 11-week-old age. Eleven-week-old DS rats with established hypertension and established cardiac hypertrophy, were randomized into two groups, and were orally given (1) vehicle or (2) linagliptin (3 mg/kg/day) by gastric gavage once a day for 4 weeks (until 15 weeks of age). DS rats fed 0.3% NaCl diet (normal-salt diet) were served as the control. After 4 weeks of the drug treatment, DS rats were anesthetized with isoflurane, arterial blood was immediately collected by cardiac puncture, and serum was collected by centrifugation and stored at −80°C until use. After perfusion with phosphate-buffered saline, the carotid artery, the thoracic aorta, and the heart were immediately excised for the measurement of various parameters, as described below. Left ventricular tissues were equally divided into three parts from apex to the base of heart; the apex, the middle, the base of heart. The real-time RT-PCR and western blot analysis was performed using the apex tissues. The Sirius red staining and immunohistochemistry were performed on paraffin embedded middle part. The DHE staining was performed on a series of cryostat sections of the base of heart.

### Echocardiography

In vivo cardiac morphology was assessed by transthoracic echocardiography (12-MHz echocardiographic probe, PHILIPS SONOS-4500), as previously described [26-28]. In brief, rats were anesthetized with isoflurane. M-mode left ventricular (LV) end-systolic and end-diastolic diameters were averaged from 3–5 beats. M-mode tracings were recorded through left ventricular anterior and posterior walls at the papillary muscle level to measure left ventricular end-diastolic dimension, left ventricular end-systolic dimension, fractional shortening, left ventricular ejection fraction, left ventricular posterior wall thickness at end diastole, and inter ventricular septum wall thickness at end diastole. LV mass index was calculated as follows: LV mass =1.04 X [(LVDd + PWd + LVPw)^3^ – LVDd^3^], according to the previous report [29]. Studies and analysis were performed by investigators in a blinded fashion.

### Vessel ring preparation and organ chamber experiments

Isometric tension was measured as previously described [30]. In brief, carotid artery from DS rats were cut into 5-mm rings with special care to preserve the endothelium, and they were then mounted in organ baths filled with modified Tyrode buffer (pH 7.4; NaCl 121 mmol/L, KCl 5.9 mmol/L, CaCl_2_ 2.5 mmol/L, MgCl_2_ 1.2 mmol/L, NaH_2_PO_4_ 1.2 mmol/L,NaHCO_3_ 15.5 mmol/L, and D-glucose 11.5 mmol/L) aerated with 95% O_2_ and 5% CO_2_ at 37°C. The preparations were attached to a force transducer, and isometric tension was recorded on a polygraph. A resting tension of 1 g was maintained throughout the experiment. Vessel rings were primed with KCl (50 mmol/L) and then precontracted with L-phenylephrine (10^−7^ mol/L). After the plateau of tension was attained, the rings were exposed to increasing concentrations of acetylcholine (10^−9^ to 10^−4^ mol/l) or sodium nitroprusside (SNP) (10^−9^ to 10^−4^ mol/L) to obtain cumulative concentration response curves.

### Measurement of blood pressure

Systolic blood pressure of conscious rats was periodically measured by tail-cuff plethysmography (BP-98A; Softron Co, Tokyo, Japan). To minimize chances for biasing of results, blood pressure measurement was performed by a technician who was blinded to the experimental groups [31]. The animals were acclimated to the measurement procedures and were prewarmed at 37°C for 20 minutes before measurement of blood pressure. Ten measurements per animal were averaged for determination of blood pressure.

### Collection of urine samples in metabolic cages

At 1 week and 3 weeks after initiation of drug treatment, the above mentioned 3 groups of DS rats were acclimatized to the metabolic cages (Techniplast 3701 M001, Buguggiate, Italy) for 24 hours, then 24-hr urine was collected with metabolic cages to measure urine volume and urinary electrolyte excretions.

### Western blot analysis

Our detailed method has been described previously [30]. Antibodies used were as follows: anti-p67^phox^ (x5000, BD Biosciences, San Jose, CA, USA), anti-p22^phox^ (x2000, Santa Cruz Biotechnology Inc, Santa Cruz, CA, USA), anti-angiotensin converting enzyme (ACE) (x2000, Abcam, Camgridge, UK). The intensity of the bands was quantified using NIH Image analysis software v1.61. In individual samples, each value was corrected for that of GAPDH.

### Measurement of tissue superoxide

Hearts and aortas removed from DS rats were immediately frozen in Tissue-Tek OCT embedding medium (Sakura Finetek, Tokyo, Japan). Dihydroethidium (DHE) was used to evaluate tissue superoxide levels in situ, as described [32]. In brief, DHE fluorescence was visualized by fluorescence microscopy using an excitation wavelength of 520–540 nm and a rhodamine emission filter. DHE fluorescence of tissue was captured with the same exposure time (1.0 s), and it was quantified using Lumina Vision. The mean fluorescence was quantified and expressed relative to values obtained from control rats. We have previously verified that DHE fluorescence obtained by our method is indeed attributed to superoxide [33].

### Histological examination and immunohistochemistry

Hearts were fixed in 4% (wt/vol.) paraformaldehyde, embedded in paraffin, sectioned at 5 μm, and stained with Sirius Red F3BA (0.5% wt/vol. in saturated aqueous picric acid; Aldrich Chemical Company, St Louis, MO, USA) for the measurement of collagen volume fraction. Cardiac interstitial fibrosis and the ratio of wall to lumen area of coronary artery were quantified, as described [34]. The positive area of fibrosis per field area was assessed by examining at least 10 fields per rat using Lumina Vision version 2.2 analysis software.

For ED-1 immunohistochemistry, cardiac sections were incubated overnight with ED1 antibody (×100; BMA Biomedicals, Switzerland) followed by Histofine simple stain Max-Po (M) (Nichirei, Biosciences, Tokyo, Japan). The number of cardiac ED-1-positive cells per mm^2^ was counted in a blinded manner by examining more than 10 fields per section using a microscope with X200 magnification. The average ED-1-positive cell number was obtained in each rat.

### Measurement of left ventricular mRNA

MCP-1 mRNA expression was quantified using real-time PCR, as described previously [35]. TaqMan primers and probes for collagen I (Rn01526721_m1), collagen III (Rn01437681_m1) and GAPDH (Rn01775763_g1) were derived from the commercially available TaqMan Gene Expression Assays (Applied Biosystem).

### Measurement of DPP-4 activity, GLP-1, and insulin

Non-fasting serum glucose, insulin, and active GLP-1 concentrations were measured by the Glucose CII-Test (Wako Pure Chemical, Osaka, Japan), the Insulin ELISA kit (Morinaga Institute of Biological Science, Inc., Yokohama, Japan), and GLP-1, the Active form Assay Kit-IBL (Immuno-Biological Laboratories, Gunma, Japan), respectively. DPP4 activity was measured using DPP4-Glo Protease Assay (Promega, Madison, WI, USA).

### Statistical analysis

All data are presented as means ± SEM. The data on blood pressure were analyzed by one-way analysis of variance (ANOVA) with repeated measures, followed by Fisher’s protected least squares difference (PLSD) test using Prism (Graph Pad Software Inc., San Diego, CA, USA). Other data were analyzed with one-way ANOVA followed by Fisher’s PLSD test, in the case of comparison among three groups. In all tests, differences were considered statistically significant at a value of P < 0.05.

## Results

### Effects of linagliptin on blood pressure of DS rats fed high-salt diet

**Figure 1:**
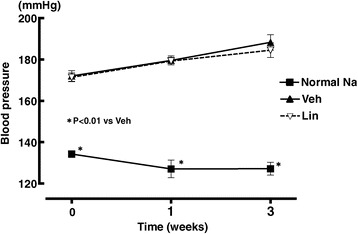
**Effect of linagliptin on blood pressure of high salt-loaded DS rats at 1 week and 3 weeks after initiation of linagliptin treatment.** Abbreviations used: Normal Na, normal salt-fed DS rats; Veh, vehicle-treated DS rats fed high-salt diet; Lin, linagliptin-treated DS rats fed high-salt diet. Each value represents mean ± SEM (n = 7 in Normal Na, n = 11 in Veh, n = 11 in Lin).

As shown in Figure 1, before initiation of linagliptin administration, DS rats fed high-salt diet for 4 weeks already developed hypertension (more than 170 mmHg). Initiation of linagliptin administration after onset of hypertension in high-salt-loaded DS rats did not significantly alter blood pressure of DS rats, throughout the treatment.

### Effects of linagliptin on cardiac weight and echocardiographic parameters

**Figure 2:**
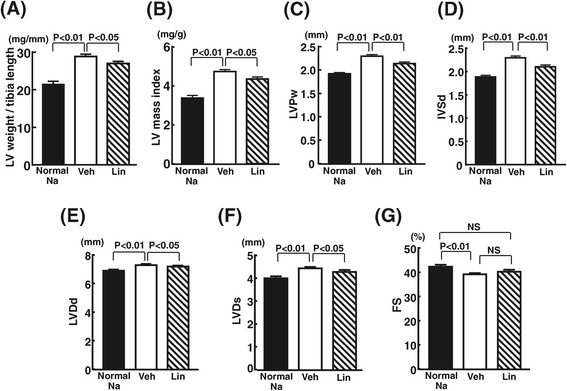
**Effect of linagliptin on cardiac weight (A) and echocardiographic parameters ((B)-(G))of high salt-loaded DS rats.** Each value represents mean ± SEM (n = 7 in Normal Na, n = 11 in Veh, n = 11 in Lin). Abbreviation used are the same as in Figure 1. LV mass index, left ventricular mass index; IVSd, interventricular septum diastolic wall thickness; LVPw, left ventricular posterior diastolic wall thickness; LVDd, left ventricular end-diastolic diameter; LVDs, left ventricular end-systolic diameter; FS, fractional shortening.

As shown in Figure 2 (A), DS rats fed high-salt diet had greater left ventricular weight than those fed normal-salt diet (P < 0.01). Linagliptin treatment significantly reduced left ventricular weight of high-salt-loaded DS rats (P < 0.05). As shown in Figure 2(B)-(F), LV mass index, left ventricular posterior diastolic wall thickness (LVPw), interventricular septum diastolic wall thickness (IVSd), left ventricular end-diastolic diameter (LVDd), and left ventricular end-systolic diameter (LVDs) of high-salt-loaded DS rats were greater than those of normal-salt fed DS rats. Linagliptin treatment significantly reduced the increase in LV mass index (P < 0.05), LVPw (P < 0.01), IVSd (P < 0.01), LVDd (P < 0.05), and LVDs (P < 0.05) of high-salt-loaded DS rats. Fractional shortening (FS) in vehicle group of high-salt fed DS rats was smaller than that in normal-salt fed DS rats (P < 0.01), while there was no difference in FS between normal-salt fed DS rats and linagliptin group of high-salt fed DS rats.

### Effects of linagliptin on body weight and organ weights of DS rats fed high-salt diet

Linagliptin treatment decreased the increase in liver weight of high-salt loaded DS rats (P < 0.05) (Table 1). There was no significant difference between vehicle group and linagliptin group of high-salt-loaded DS rats, regarding body weight, lung weight, or kidney weight (Table 1).

**Table 1:** **Effects of linagliptin on body weight and various organ weights in salt-loaded DS rats**

		**High Na**	
	**Normal Na**	**Veh**	**Lin**
Body weight (g)	378 ± 9	367 ± 5	353 ± 5
Lung weight/tibia length (mg/mm)	37 ± 1	39 ± 2	37 ± 1
Liver weight/tibia length (mg/mm)	320 ± 4^#^	344 ± 8	324 ± 6^#^
Kidney weight/tibia length (mg/mm)	70 ± 1^*^	95 ± 2	92 ± 1

Normal Na, normal-salt diet-fed DS rats, Veh, vehicle-treated DS rats fed high-salt diet, Lin, linagliptin-treated DS rats fed high-salt diet. Values are the means ± SEM (n = 7-11). Statistical analysis was performed by one-factor analysis of variation (ANOVA) followed by post hoc Fisher’s protected least significant difference test. ^#^P < 0.05,

^*^P < 0.01 vs Veh.

### Effects of linagliptin on cardiac fibrosis, inflammation, and coronary arterial remodeling of DS rats fed high-salt diet

**Figure 3:**
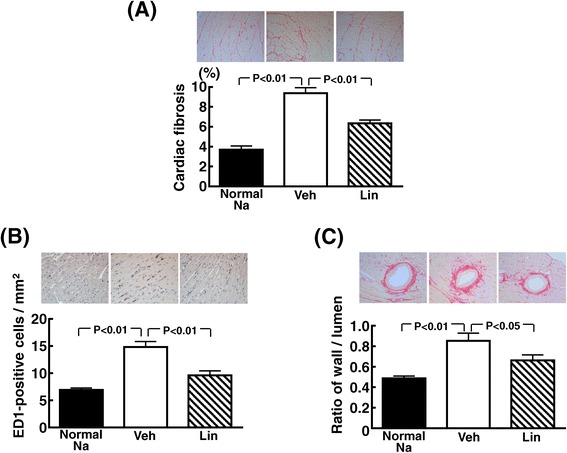
**Effect of linagliptin on cardiac interstitial fibrosis (A), ED-1-positive cell numbers (B), and coronary arterial remodeling (C) of high salt-loaded DS rats.** Each value represents mean ± SEM (n = 5-7 in Normal Na, n = 11 in Veh, n = 11 in Lin). Abbreviations used are the same as in Figure 1. Upper panels in **(A)**, **(B)**, and **(C)** indicate representative photomicrographs of cardiac sections stained with Sirius red, ED-1, and Sirius red, respectively.

As shown in Figure 3, high-salt-loaded DS rats exhibited greater cardiac interstitial fibrosis (P < 0.01), greater ED1-positive cell (macrophage) infiltration (P < 0.01), and greater ratio of wall to lumen of coronary artery (P < 0.01) than those fed normal-salt diet. Linagliptin treatment significantly reduced the increase in cardiac interstitial fibrosis (P < 0.01), cardiac ED1-positive cell number (P < 0.01), and ratio of wall to lumen of coronary artery (P < 0.05) in high-salt-loaded DS rats.

### Effects of linagliptin on cardiac oxidative stress, p67^phox^, angiotensin-converting enzyme, and MCP-1 or collagen mRNA

**Figure 4:**
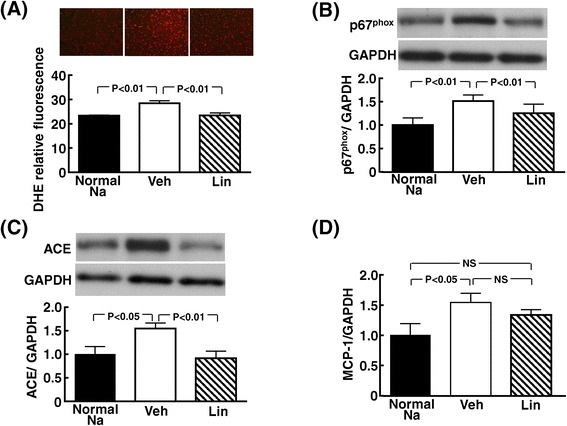
**Effect of linagliptin on cardiac oxidative stress (A), p67** ^**phox**^ **(B), ACE (C), and MCP-1 mRNA (D) of high salt-loaded DS rats.** Each value represents mean ± SEM (n = 7 in Normal Na, n = 11 in Veh, n = 11 in Lin). Abbreviations used are the same as in Figure 1. NS, not significant between groups. Upper panels in **(A)** indicate representative photomicrographs of cardiac sections stained with dihydroethidium. Upper panels in **(B)** and **(C)** indicate representative western blot of p67^phox^ and ACE, respectively. In **(D)**, MCP-1 mRNA levels in individual rats were corrected for GAPDH mRNA levels.

As shown in Figure 4, linagliptin treatment significantly ameliorated the increase in cardiac superoxide (P < 0.01), cardiac NADPH oxidase subunit p67^**phox**^ (P < 0.01), and cardiac ACE levels (P < 0.01) of high-salt loaded DS rats.

Linagliptin treatment tended to reduce cardiac MCP-1 mRNA levels in high-salt fed DS rats, although the difference did not reach statistical significance (Figure 4 (D)). Cardiac collagen I mRNA levels were 1.00 ± 0.22, 1.68 ± 0.23, and 1.35 ± 0.20 and cardiac collagen III mRNA levels were 1.00 ± 0.09, 2.18 ± 0.33, and 1.61 ± 0.17, in normal-salt fed DS rats, vehicle-treated high-salt fed DS rats, and linagliptin-treated high-salt fed rats, respectively. There was a trend for reduction of collagen I and collagen III mRNA levels by linagliptin, although the difference did not reach statistical significance.

### Effects of linagliptin on vascular function and oxidative stress of DS rats

**Figure 5:**
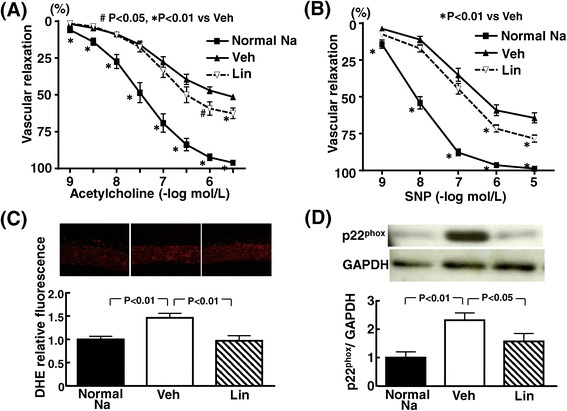
**Effect of linagliptin on vascular relaxation induced by acetylcholine (A) and SNP (B), vascular superoxide (C), and vascular p22phox (D) of high salt-loaded DS rats.** Each value represents mean ± SEM (n = 7 in Normal Na, n = 9-11 in Veh, n = 11 in Lin). Abbreviations used are the same as in Figure 1. NS, not significant between groups. Upper panels in **(C)** and **(D)** indicate representative photomicrographs of aortic sections stained with dihydroethidium and representative western blot bands, respectively.

As shown in Figure 5, linagliptin significantly ameliorated the impairment of acetylcholine-induced vascular relaxation and that of SNP-induced vascular relaxation of high-salt loaded DS rats. As shown in Figure 5 (C), linagliptin reduced the increase in vascular superoxide of high-salt loaded DS rats (P < 0.01). Figure 5 (D) indicates that linagliptin significantly attenuated the increase in vascular p22^phox^ in high-salt loaded DS rats (P < 0.05).

### Effects of linagliptin on non-fasting serum DPP-4 activity, GLP-1 concentrations, insulin, and glucose of DS rats

**Figure 6:**
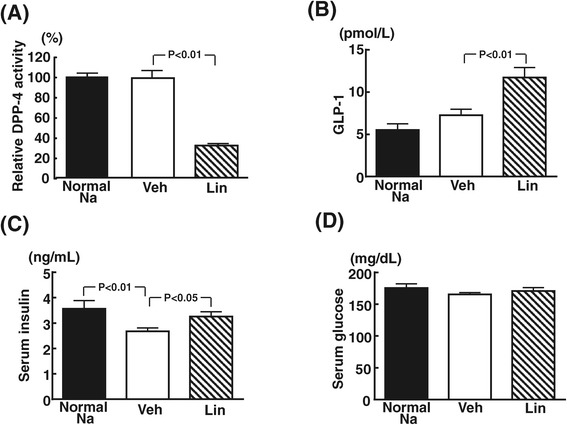
**Effect of linagliptin on serum DPP4 activity, GLP-1, insulin, and glucose levels of high salt-loaded DS rats.** Each panel indicates relative DPP-4 activity **(A)**, GLP-1 **(B)**, serum insulin **(C)**, and serum glucose **(D)**. Abbreviations used are the same as in Figure 1. Each value represents mean ± SEM (n = 7 in Normal Na, n = 11 in Veh, n = 10 in Lin).

As shown in Figure 6, linagliptin significantly decreased non-fasting serum DPP-4 activity of DS rats by 67.7% (P < 0.01), which was accompanied by the significant increase in serum GLP-1 concentrations by 1.6-fold (P < 0.01) and the slight but significant increase in non-fasting serum insulin (P < 0.05). However, there was no difference in non-fasting blood glucose levels between normal-salt and high-salt fed DS rats, and linagliptin did not alter non-fasting blood glucose levels in high-salt fed DS rats.

### Effects of linagliptin on urine volume and urinary electrolytes

Table 2 indicates the data on 24-hour urine sample collected with metabolic cages at 1 and 3 weeks after initiation of linagliptin treatment. At both time points, high-salt loaded DS rats displayed greater urine volume and greater excretions of urinary sodium, chloride, and potassium than normal-salt-fed DS rats. Linagliptin did not significantly affect 24-hour urine volume and 24-hour excretions of urinary sodium, chloride, and potassium in high-salt loaded DS rats, throughout the treatment. Food intake and water intake in DS rats were not altered by linagliptin treatment, compared with vehicle treatment.

**Table 2:** **Effects of linagliptin on 24-hr-food intake, water intake, urine volume, and urinary electrolyte excretions of high-salt loaded DS rats at 1 week and 3 weeks after initiation of linagliptin treatment**

	**1 wk after the treatment**			**3 wk after the treatment**		
	**Normal Na**	**Veh**	**Lin**	**Normal Na**	**Veh**	**Lin**
Food intake (g/day)	24.0 ± 2.0	24.3 ± 1.1	22.3 ± 0.6	28.2 ± 1.8^#^	22.2 ± 1.5	22.8 ± 0.9
Water intake (ml/day)	19.7 ± 5.2^*^	84.3 ± 5.9	84.9 ± 4.0	22.8 ± 3.3*	81.3 ± 3.6	81.8 ± 3.4
Urine volume (g/day)	13.5 ± 4.4*	68.6 ± 3.8	65.1 ± 2.6	14.2 ± 1.8*	72.4 ± 3.7	70.4 ± 2.9
Urinary Na (mEq/day)	0.5 ± 0.03*	28.7 ± 1.1	28.7 ± 0.6	0.4 ± 0.03*	29.0 ± 1.0	29.0 ± 1.2
Urinary Cl (mEq/day)	0.6 ± 0.02*	28.4 ± 1.1	28.5 ± 0.7	0.6 ± 0.04*	28.4 ± 0.9	26.6 ± 2.6
Urinary K (mEq/day)	2.9 ± 0.2*	4.0 ± 0.2	4.0 ± 0.1	3.3 ± 0.1^#^	3.9 ± 0.1	4.0 ± 0.2

Abbreviations used are the same as in Table 1. Values are the means ± SEM (n = 7-11). Statistical analysis was performed by one-factor analysis of variation (ANOVA) followed by post hoc Fisher’s protected least significant difference test.

^#^P < 0.05, ^*^P < 0.01 vs Veh.

## Discussion

The main purpose of this work was to determine whether initiation of DPP-4 inhibitor linagliptin administration after onset of hypertension and cardiac hypertrophy can exert direct beneficial effects on cardiovascular injury induced by salt-sensitive hypertension. To address this issue, in this work, we used DS rats which are a useful model of salt-sensitive hypertension and display insulin resistance and no fasting hyperglycemia [36-39]. Furthermore, we used linagliptin in the present study, because linagliptin has a unique xanthine-based structure and takes the advantage of being used in patients with renal dysfunction without dose adjustment in contrast to other approved DPP-4 inhibitors [22,23]. Moreover, linagliptin significantly improves glycemic control and is well tolerated in patients with type 2 diabetes complicated by hypertension [40] and is hypothesized to have cardiovascular benefits in type 2 diabetic patients [41]. The major findings of this study were that linagliptin directly ameliorated cardiac hypertrophy, inflammation, and fibrosis, coronary arterial remodeling, and vascular endothelial dysfunction in salt-sensitive hypertensive rats, and these protective effects of linagliptin were associated with the attenuation of cardiac and vascular oxidative stress and cardiac ACE. Thus, our present work provides a novel insight into the mechanism underling cardiovascular protection by DPP-4 inhibition. Moreover, our present findings provide important clinical implications in the management of diabetes complicated with hypertension, since initiation of linagliptin administration after onset of hypertension was shown to protect against cardiovascular injury.

Type 2 diabetes and hypertension frequently coexist in the same patients. Importantly, hypertensive patients with coexisting diabetes are often characterized by salt-sensitive hypertension. Furthermore, salt-sensitive hypertension is shown to have greater risk for cardiovascular events than salt-resistant hypertension [17-21]. Therefore, any effects of DPP-4 inhibition on blood pressure and cardiovascular injury in the setting of salt-sensitive hypertension are of particular interest. However, the impact of DPP-4 inhibition on salt-sensitive hypertension remains to be defined. These encouraged us to investigate the effect of linagliptin on blood pressure and cardiovascular injury in salt-sensitive hypertensive rats. There has been uncertainty about the effect of DPP-4 inhibitors on blood pressure. It has been reported that DPP-4 inhibition significantly reduces blood pressure in spontaneously hypertensive rats (SHR) [42,43] and very slightly lowers blood pressure in nondiabetic patients [44]. On the other hand, other studies show that DPP-4 inhibitor has no effect on blood pressure in renovascular hypertensive rats [45], increases blood pressure in SHR [46] and diminish hypotensive effects of high-dose of ACE inhibitor in subjects with metabolic syndrome [47]. Thus, the effect of DPP-4 inhibition on blood pressure appears to be dependent on type of hypertension or experimental conditions. In the present study, initiation of linagliptin administration after onset of hypertension did not significantly alter blood pressure of salt-sensitive hypertensive rats. Moreover, our data on 24-hr urine collected with metabolic cage indicated no significant alteration of urine volume and urinary sodium, chloride, and potassium excretions by linagliptin in DS rats, showing no significant natriuretic effects of linagliptin in DS rats. Collectively, our present work supported the notion that the protective effects of linagliptin against cardiovascular injury in DS rats were mediated by its direct pleiotrophic effects, independently of blood pressure.

Oxidative stress [48-50] and inflammation [51] play a key role in the pathogenesis of cardiovascular disease, including cardiac hypertrophy and remodeling, vascular endothelial dysfunction, and atherosclerosis. Moreover, oxidative stress and inflammation are shown to be involved in cardiovascular injury in DS rats [27]. Of note, in the present study, linagliptin markedly attenuated oxidative stress in cardiac and vascular tissues of DS rats as shown by the reduction of cardiovascular superoxide. Furthermore, linagliptin significantly reduced cardiac macrophage infiltration in DS rats, as shown by the decrease in ED1-positive cell (a marker of macrophage) by linagliptin. Thus, the protective effects of linagliptin against cardiovascular injury in DS rats appear to be at least in part mediated by the significant decrease in cardiovascular oxidative stress and inflammation. Interestingly, linagliptin significantly reduced cardiac p67^phox^ and vascular p22 ^phox^ of DS rats. p67^phox^ and p22 ^phox^ are key subunits of NADPH oxidase generating superoxide [52]. Therefore, the attenuation of cardiovascular oxidative stress by linagliptin might be partially attributed to the reduction of p67^phox^ or p22 ^phox^.

A large number of clinical and experimental studies provide the solid evidence that ACE plays a central role in the pathogenesis of cardiac hypertrophy and fibrosis and heart failure [1]. A subdepressor dose of ACE inhibitor administration significantly attenuates cardiac oxidative stress and improves cardiac function in DS rats without affecting blood pressure, indicating that cardiac ACE is involved in cardiac oxidative stress and cardiac dysfunction independently of blood pressure [53-55]. In fact, in clinical practice, ACE inhibitors are well established to be effective for treatment of cardiovascular disease in a broad range of high-risk patients including diabetic patients [1,56]. There have been many reports showing that cardiac ACE plays a causal role in cardiac hypertrophy and remodeling in DS rats [26,53,57]. In the present study, it is noteworthy that the increased cardiac ACE in DS rats was significantly reduced by linagliptin treatment. Collectively, the cardioprotective effects of linagliptin observed in this work seems to be at least in part mediated by the reduction of cardiac ACE.

In our present study, expectedly, linagliptin treatment of DS rats significantly reduced serum DPP-4 activity, being accompanied by the significant increase in circulating GLP-1. Despite the significant increase in serum GLP-1 and insulin by linagliptin, blood glucose levels were not significantly altered by linagliptin. No alteration of blood glucose by linagliptin is in good agreement with the previous findings that the significant improvement of insulin resistance by GLP-1 or GLP-1 receptor analogue in DS rats did not change blood glucose levels in DS rats [58], and seems to be explained by the fact that DS rats exhibit insulin resistance but no fasting hyperglycemia [36-39]. Importantly, the increase in circulating GLP-1 with DPP-4 inhibition is much smaller than that with exogenous GLP-1 administration. DPP-4 inhibitors are likely to affect other peptides than GLP-1, since DPP-4 is a multifunctional enzyme and can cleave a number of other substrates than GLP-1, and DPP-4 inhibitors are proposed to potentially confer cardiovascular protective effects through GLP-1- independent mechanism [6,8,59-61]. Interestingly, previous report [62] indicated that chronic infusion of exogenous GLP-1 significantly lowered blood pressure of DS rats through natriuresis, and these findings [62] are different from our present observations indicating no alteration of blood pressure and no increase in urinary sodium excretion by linagliptin in DS rats. Collectively, these findings support the notion that DPP-4 inhibitor potentially has different mode of action from GLP-1 or GLP-1 agonist and cardiovascular protective effect of linagliptin observed in this study might be partially mediated by GLP-1-independent mechanism. However, further study is needed to elucidate the precise mechanism underling the cardiovascular protective effects of linagliptin in salt-sensitive hypertension.

### Study limitation

There are several study limitations in this study. First, as cardiac function of DS rats were investigated at the stage of no cardiac diastolic dysfunction (15 weeks of age), our present work did not allow us to determine whether linagliptin can finally improve cardiac diastolic dysfunction of DS rats. Previous study [10] investigating the preventive effect of linagliptin in insulin-resistant Zucker obese rats shows that linagliptin prevented the development of cardiac diastolic dysfunction despite no attenuation of cardiac oxidative stress, cardiac hypertrophy or fibrosis. Therefore, future study is required to elucidate the therapeutic effect of longer term administration of DPP4 inhibitor on cardiac diastolic dysfunction. Furthermore, in the present study, as the difference in echocardiographic parameters between the groups was small, more sensitive method such as hemodynamic study is needed to validate the improvement of cardiac function by linagliptin. Second, the present work provided no detailed mechanism underlying the attenuation of cardiac fibrosis, inflammation, and oxidative stress by linagliptin. Particularly, further analysis of oxidative stress marker is required to confirm the attenuation of oxidative stress by linagliptin. Third, it cannot be ruled out that the improvement of acetylcholine-induced vascular relaxation by linagliptin might be attributed to the improvement of vascular smooth muscle cell function rather than the improvement of endothelial function, since linagliptin also ameliorated the impairment of smooth muscle cell-dependent vascular relaxation. However, in vitro study shows that linagliptin specifically suppresses endothelial cell damage and reduce endothelial oxidative stress [14]. Finally, direct blood pressure measurement was not performed in the present study. Accordingly, it cannot be completely excluded that linagliptin might reduce blood pressure of DS rats to a small extent, although the tale-cuff method is a popular and established method for determination of blood pressure [31].

## Conclusions

In conclusion, our work provided the evidence that DPP-4 inhibitor linagliptin protected against cardiovascular injury in salt-sensitive hypertensive rats, independently of blood glucose or blood pressure. These beneficial effects of linagliptin were associated with the attenuation of oxidative stress and cardiac ACE. Since linagliptin initiated after onset of hypertension was shown to exert positive therapeutic cardiovascular effects, our work highlights linagliptin as potentially a promising therapeutic agent for treatment of macrovascular disease in patients with coexisting diabetes and hypertension. However, further clinical trials investigating the effect of DPP-4 inhibition on cardiovascular outcome is required to define our proposal.

## Abbreviations

ACE: Angiotensin converting enzyme
DPP-4: Dipeptidylpeptidase-4
DS rat: Dahl salt-sensitive hypertensive rats
GLP-1: Glucagon-like peptide-1
LV: Left ventricular
SNP: Sodium nitroprusside
